# Intense selective hunting leads to artificial evolution in horn size

**DOI:** 10.1111/eva.12358

**Published:** 2016-01-29

**Authors:** Gabriel Pigeon, Marco Festa‐Bianchet, David W. Coltman, Fanie Pelletier

**Affiliations:** ^1^ Département de Biologie and Centre d’Études NordiquesUniversité de SherbrookeSherbrookeQCCanada; ^2^ Département de BiologieCanada Research Chair in Evolutionary Demography and ConservationUniversité de SherbrookeSherbrookeQCCanada; ^3^ Department of Biological SciencesUniversity of AlbertaEdmontonABCanada

**Keywords:** conservation biology, contemporary evolution, quantitative genetics

## Abstract

The potential for selective harvests to induce rapid evolutionary change is an important question for conservation and evolutionary biology, with numerous biological, social and economic implications. We analyze 39 years of phenotypic data on horn size in bighorn sheep (*Ovis canadensis*) subject to intense trophy hunting for 23 years, after which harvests nearly ceased. Our analyses revealed a significant decline in genetic value for horn length of rams, consistent with an evolutionary response to artificial selection on this trait. The probability that the observed change in male horn length was due solely to drift is 9.9%. Female horn length and male horn base, traits genetically correlated to the trait under selection, showed weak declining trends. There was no temporal trend in genetic value for female horn base circumference, a trait not directly targeted by selective hunting and not genetically correlated with male horn length. The decline in genetic value for male horn length stopped, but was not reversed, when hunting pressure was drastically reduced. Our analysis provides support for the contention that selective hunting led to a reduction in horn length through evolutionary change. It also confirms that after artificial selection stops, recovery through natural selection is slow.

## Introduction

Human activities such as habitat modifications, expanding road networks, overexploitation and climate change affect animal populations. While the demographic impacts of humans on wild species are clear, their evolutionary impacts are debated (Loehr et al. 2007; Hard et al. 2008). Intense exploitation by humans may outpace (Darimont et al. 2009) or oppose (Carlson et al. 2007) the selective effects of natural predators, potentially leading to evolutionary changes in behaviour, phenotype or life history (Hard et al. 2008; Devine et al. 2012). van Wijk et al. (2013) showed that selective harvesting of guppies (*Poecilia reticulata*) led to changes in size and in the frequency of alleles associated with size in just two generation. Human‐induced evolution may also impair population persistence or prevent recovery (Swain et al. 2007; Uusi‐Heikkilä et al. 2015). While numerous studies of fishes report evidence of evolution induced by intense harvest (reviewed in Hutchings and Fraser 2008), evidence for evolution through selective harvest in terrestrial species remains scarce and controversial (Coltman et al. 2003; Garel et al. 2007; Mysterud 2011; Traill et al. 2014), partly because the statistical techniques used to quantify evolutionary changes using pedigrees in earlier studies have been questioned (Postma 2006; Hadfield et al. 2010).

Trophy hunting can be an important component of many conservation programs (Leader‐Williams et al. 2001), and its economic revenues are partly driven by expectation of large trophy size (Festa‐Bianchet and Lee 2009; Crosmary et al. 2013). In most of Canada, sport harvest of mountain sheep (*Ovis canadensis* and *O. dalli*) rams is based on a phenotypic definition of minimum horn curl that establishes whether or not a ram can be shot, with an unlimited number of permits available to resident hunters (Festa‐Bianchet et al. 2014). In wild sheep, horn size is a key determinant of success in male‐male competition over breeding opportunities (Coltman et al. 2002). Artificial selection favoring shorter horns through hunting mortality, however, sets in 2–3 years before natural selection favoring longer horns through reproductive success (Coltman et al. 2002). Multiple studies report that males with fast‐growing horns, that would enjoy high mating success at 8–10 years of age, are harvested at 4–7 years, conferring a reproductive advantage to small‐horned males that, in the absence of size‐selective harvests, would normally be outcompeted (Festa‐Bianchet et al. 2004, 2014; Loehr et al. 2007; Hengeveld and Festa‐Bianchet 2011; Douhard et al. In Press).

One approach to study evolution in nature, often referred to as the animal model, involves mixed models combining a pedigree with data on phenotype and environmental conditions to estimate genetic parameters (Kruuk 2004). Using this approach, Coltman et al. (2003) used a pedigree up to six generation deep to report a decline in estimated breeding values (EBV) of horn length and body mass in bighorn rams over 30 years, suggesting an evolutionary response to size‐selective harvests. Their analyses, however, were criticised for not adequately accounting for environmental effects on phenotype, for the error in estimation of breeding values and for the effect of drift; possibly leading to exaggerated estimates of evolutionary change (Postma 2006; Hadfield et al. 2010). Hence, the importance of evolution in the observed change in phenotype following selective harvesting is still debated.

A recent paper used data from the individually monitored population of bighorn sheep of Ram Mountain to parameterise an Integral Projection Model and show a decline in body mass, but argued that the phenotypic response to harvest was only demographic (Traill et al. 2014). The statistical criticisms and alternative analyses listed above cast doubt on the conclusion that selective hunting could lead to evolutionary changes. Coltman et al. (2003) drew that conclusion after analysing data for the only sport‐hunted population of ungulates for which a pedigree and horn measurements are available (Pelletier et al. 2012). By extension, these criticisms also question phenotype‐based studies that reported long‐term trends consistent with an evolutionary impact of selective hunting (Garel et al. 2007; Hengeveld and Festa‐Bianchet 2011). A clear understanding of the importance of evolutionary change due to selective harvesting is of critical importance to those responsible for managing harvested wild populations (Allendorf and Hard 2009). A reanalysis of the Ram Mountain data is therefore warranted, particularly because the 10‐fold decline in harvests after 1996 provides an opportunity to test the impacts of changes in harvest pressure on trait evolution (Douhard et al. In Press).

Here, we use a Bayesian animal model to analyse an expanded database on bighorn sheep from Ram Mountain, adding 9 years of data to those available to Coltman et al. (2003) and taking into account subsequent statistical criticisms (Postma 2006; Hadfield et al. 2010). We also compare a period of intense harvest with a period when harvest was first dramatically reduced, then stopped. This allowed us to compare temporal trends in genetic values under heavy and very light artificial selection. To maximise the use of phenotypic information, we considered data on male and female traits using a multivariate model. Genetic correlations have already been established among some of these traits (Poissant et al. 2012), and proper estimation of breeding values must account for genetic covariance (Wolak et al. 2015). By including phenotypic data on females we could also compare temporal changes in traits that are (male horn base and female horn length) and are not (female horn base) genetically correlated to male horn length (Poissant et al. 2012). We expected to see temporal changes in EBV in male horn length only under heavy harvest. Male horn base circumference is particularly interesting because it is correlated with horn length and likely affects male‐male competition by contributing to horn mass, but is not a direct target of selective hunting. We expected strong selective effects on male horn length, the trait most directly related to the legal definition of harvestable ram (Festa‐Bianchet et al. 2014). We expected a response similar to male horn length for male horn base and female horn length given their strong genetic correlations with male horn length, and no response in female horn base, which has a weak genetic correlation with male horn length (genetic correlations of 0.72, 1 and −0.28 respectively; Poissant et al. 2012). To compare our results with previous studies on this population, we also built animal models using univariate one‐sex (with phenotypic data on males only) and two‐sex (phenotypic data from both sexes) approach. Univariate models are also less prone to problems when fitted with limited data given their simpler structure (Wilson et al. 2010).

## Material and methods

### Study population and phenotypic data

Bighorn sheep at Ram Mountain, Alberta, Canada are intensively monitored. The study area is 30 km east of the Rockies (52°8′N, 115°8′W, elevation 1082–2173 m), on a mountainous outcrop dominated by cliffs, rock scree and alpine meadows. Since 1972, sheep have been marked with ear tags and collars. Each year, between May and September, sheep were repeatedly captured in a corral trap baited with salt. Rams were captured on average 2.6 times per year. At each capture, horn length in cm was measured along the outside curvature with a flexible tape. To reduce the potential measurement error caused by horn wear or breakage, we used the longest horn in analyses. Horn base circumference was also measured in cm, and we analysed the mean of the left and right measurements. Nearly all individuals (95%) were first captured as lambs or yearlings, so their exact age was known. For the others, age was determined using horn annuli.

The study population was hunted until 2011 based on a morphological definition of ‘legal’ ram. From late August to October, rams were at a risk of being shot only if they met that definition, which specified a minimum degree of horn curl and was correlated with horn length (Festa‐Bianchet et al. 2014). Artificial selection through hunting, however, changed over time. In 1996, the minimum horn curl of a ‘legal’ ram was increased from 4/5 to full curl (Fig. S1). This change, implemented at a time when horn size had declined (Coltman et al. 2003), drastically decreased the harvest, with only four rams shot in the following 15 years. Mean harvest was 2.26 rams/year in 1973–1995 and only 0.27 rams/year in 1996–2010. Hunting was closed in 2011. Therefore, we compared trends in the EBV of morphological traits for cohorts of 1973–1996 (referred to as the hunted period) and 1996–2011 (non‐hunted period; see below). Based on the average age of fathers at Ram Mountain (7.3 years), we monitored 3.3 generations under strong artificial selection followed by 2.2 generations under natural selection.

We first adjusted all traits to September 15 using a mixed model approach (Martin and Pelletier 2011). As adult females and adult males display different growth curves, we used sex‐specific linear models to account for capture date and fitted one model per year to allow for environmental variability. Trait was fitted as a function of the square root of Julian date, considering May 25th as day 1. With this modelling approach, individual identity can be used to estimate an individual intercept and slope, providing a more accurate standardization than classical least square regression (Martin and Pelletier 2011). The procedure was used for horn length and horn base. A total of 2295 adjusted phenotypic measurements where obtained from 510 females and 497 males.

### Pedigree reconstruction

Since 1972, maternities were assigned from observation of suckling behaviour. Since 1988, DNA samples have allowed the assignation of paternities based on 26 microsatellite loci with a confidence threshold of 95% using CERVUS (Coltman et al. 2005). The pedigree in 2014 contained 864 maternal links involving 254 dams and 528 paternal links involving 79 sampled and 37 unsampled sires, the latter identified using COLONY (Jones and Wang 2010). Unsampled sires include rams that died before we began sampling for DNA and immigrants that are on Ram Mountain only for the rut.

### Quantitative genetic analyses

Analyses of horn base include phenotypic data of individuals aged 2–10 years between 1975 and 2013. For horn length, however, we only included data for sheep aged 2–4 years. Horns frequently break, and the chance of horn damage increases with age. Many old males have broken horns, missing up to the first 2 years of growth. Our data suggest that by 4 years of age ewes have reached 97% of the horn length they will have at age 8 (including the effect of breakage), and rams 73%. In addition, after age 4 the sample of rams is biased because those with longer horns are removed by hunters (Coltman et al. 2002). To reduce the importance of maternal effects on phenotypes (Wilson et al. 2005), analyses excluded phenotypic data of lambs and yearling (Réale et al. 1999; Wilson, Kruuk, and Coltman 2005).

The multivariate animal model was fitted using four traits: male horn length, female horn length, male horn base and female horn base. Phenotypic variance was then partitioned into its components, including additive genetic variance. The model also included sheep identity, year of measurement and year of birth as random effects to assess the amount of variance due to permanent, yearly and cohort environmental effects respectively. Including year of measurement and year of birth as random effects accounts for both short‐ and long‐term environmental effects, including changes in density, weather and forage quality. The year effect is necessary to obtain unbiased estimates of breeding value but it may also partly absorb temporal genetic trends, making this analysis conservative. Maternal identity was not included since the exclusion of lambs and yearlings minimized maternal effects. Age was included as a categorical fixed effect. To compare our results with previously published studies on this population we also examined univariate animal models (see supplementary material). We tested univariate models using male phenotype only (SI 2) to obtain results comparable to Coltman et al. (2003). We also fitted univariate models including both male and female phenotype to increase power (SI 3). These models are further described and their results presented in the supplementary material (SI 2–3).

The animal model estimates the breeding value of each individual. To correctly estimate breeding values and their associated error (Hadfield et al. 2010), the model was fitted using a Bayesian method with MCMCglmm version 2.21. We used a multivariate inverse‐Wishart prior to obtain the most objective results possible. Models were run using two chains for 8 500 000 iterations, with a thinning of 75 000 and a burn‐in of 1 000 000 iterations. A sensitivity analysis evaluated the robustness of the model to different prior specifications (Fig. S2).

### Temporal change in estimated breeding value

We compared temporal trends in EBV to those obtained based on different models of evolutionary change. For each realization of the MCMC chain of the animal model, we calculated mean EBV by cohort and the slope in mean EBV as a function of cohort (ße) for both the hunted and not‐hunted periods to obtain a posterior distribution of slopes. We compared this distribution to the posterior distribution of slopes of alternative models, which included no change, drift, stasis and expected evolutionary response. To compare the posterior distributions of slopes, we subtracted each realization of the posterior distribution of the alternative model to that of the distribution ße, obtaining a distribution of differences. From this distribution, we can obtain the mean difference between the expected and observed distributions as well as the confidence interval of the difference.

First, as done previously by Coltman et al. (2003), we compared slopes in EBV to 0. Second, following Hadfield et al. (2010), we compared the slopes in EBV to those obtained from simulated drift. To do so, we simulated random breeding values down the pedigree for each of the 1000 posterior samples of the animal model based on the estimated additive genetic variance. We then fitted a linear regression to the cohort mean of these random breeding values to obtain the slopes due to drift for each posterior sample. Third, we compared observed change in estimated breeding value to stasis (Hunt 2007), a pattern likely to occur under stabilizing selection. To simulate stasis, mean cohort breeding values were randomly drawn from a normal distribution with a mean of 0 and a variance equal to the observed variance in mean cohort EBV.

We also compared observed change in EBV to the response to selection predicted by the secondary theorem of selection (Morrissey et al. 2012). This theorem states that change should be equal to the additive genetic covariance between the trait of interest and relative fitness. We used longevity as a fitness measure, which we divided by mean cohort longevity to obtain relative fitness. We used longevity rather than reproductive success because molecular assignments of paternities only began in 1988. We then fitted separate bivariate animal models of trait and fitness for each of the studied traits. The predicted response to selection was then extracted from the G matrix and divided by the mean generation time (7.3 years, the average age of fathers in our population) to obtain a predicted change per year (Table S1). Predicted response to selection could only be estimated for the hunted period due to the limited number of individuals of known longevity born after hunting pressure was reduced. The proportion of iterations for which the slope for the estimated breeding value (ße) is lower than that of the random breeding value (ßr) was also calculated to estimate the probability that the trend was not caused solely by drift.

## Results

### Animal model analyses

Estimates of variance components and heritability for horn length and base (Table 1) showed that heritability was >0 for all traits. The trait with the highest posterior mode for heritability was male horn length, followed by male horn base, female horn length and female horn base. Permanent environmental effects explained much of the variance in female but not in male traits. Cohort always explained a significant part of phenotypic variance, while the effects of year and permanent environment varied among traits (Table 1). Confirming previous analyses (Poissant et al. 2012), genetic correlations between male horn length, female horn length and male horn base were high while female horn base had low genetic correlation with other traits (Table 2).

**Table 1:** Variance components and heritability of horn length and horn base in bighorn sheep at Ram Mountain, Canada, according to multivariate animal models. The posterior mode of the proportion of phenotypic variance explained by each component is followed by the 95% Bayesian posterior interval of highest density in parentheses

	Horn length male	Horn length female	Horn base male	Horn base female
*h* ^2^	0.397 (0.203–0.534)	0.223 (0.090–0.446)	0.250 (0.119–0.413)	0.265 (0.148–0.335)
ID	0.025 (0.003–0.211)	0.376 (0.203–0.540)	0.098 (0.016–0.268)	0.171 (0.110–0.265)
yr	0.110 (0.039–0.168)	0.022 (0.010–0.052)	0.193 (0.109–0.289)	0.161 (0.112–0.268)
Cohort	0.363 (0.211–0.528)	0.149 (0.071–0.286)	0.203 (0.097–0.354)	0.212 (0.107–0.291)

*h* ^2^ refers to the narrow‐sense heritability, ID refers to the proportion of phenotypic variance explained by permanent environment (identity of the sheep), yr refers to the proportion of phenotypic variance explained by year of measurement and cohort refers to the proportion of phenotypic variance explained by year of birth.

**Table 2:** Genetic correlations and covariance matrix for horn size in bighorn sheep. Values on the diagonal (grey shading) are posterior modes of genetic additive variance

	Hl‐M	Hl‐F	Hb‐M	Hb‐F
Hl‐M	17.884 (9.82–25.881)	0.921 (0.557–0.981)	0.878 (0.729–0.959)	0.189 (−0.285–0.538)
Hl‐F	5.345 (1.928–8.144)	1.622 (0.748–3.963)	0.799 (0.275–0.939)	0.368 (−0.063–0.610)
Hb‐M	5.435 (2.797–9.666)	1.274 (0.318–2.881)	2.915 (1.124–4.485)	0.286 (−0.203–0.656)
Hb‐F	0.070 (−0.542–1.059)	0.187 (−0.062–0.481)	0.182 (−0.164–0.508)	0.183 (0.119–0.270)

Values below the diagonal are the posterior modes of genetic covariance between traits: male horn length (HL‐M), female horn length (HL‐F), male horn base (HB‐M) and female horn base (HB‐F). Values above the diagonal are the posterior modes of genetic correlations. Values in parentheses represent the 95% Bayesian posterior interval of highest density.

### Temporal changes in EBV

**Figure 1:**
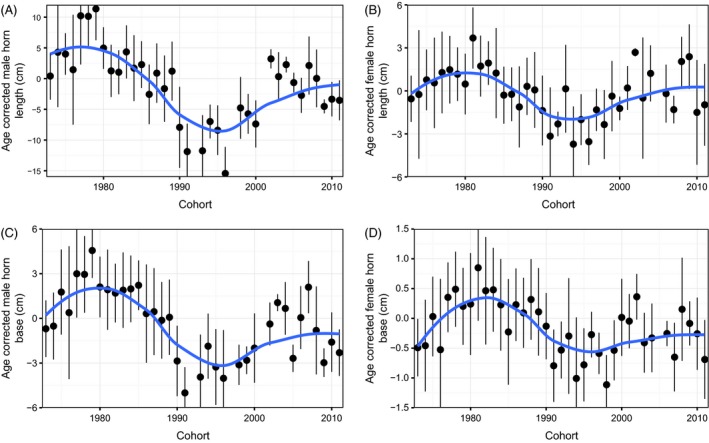
Temporal trends in age‐corrected phenotypic traits for bighorn sheep cohorts born at Ram Mountain, Canada, between 1973 and 2011. Panels show mean (A, B) horn length and (C, D) horn base in cm. Black dots and error bars represent the cohort average (±1 SD) phenotype after correcting for age. Smooths (blue line) were fitted using loess.

**Figure 2:**
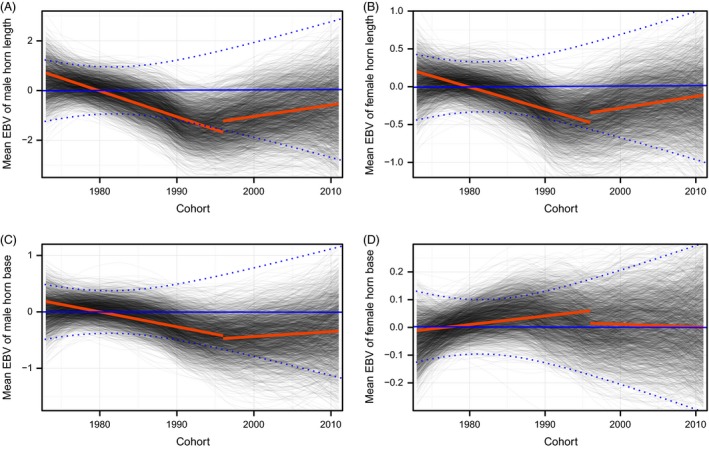
Changes in mean estimated breeding values (EBV) for bighorn sheep cohorts born at Ram Mountain between 1973 and 2011, according to a multivariate model. Panels present the EBV of (A, B) horn length and (C, D) horn base in cm. The left column shows results for males and the right column for females. Each grey line represents the average estimated breeding value through time for one iteration of the MCMC chain of the animal model using loess. Red lines represent the posterior mean trend using linear regression for the hunted and non‐hunted period. The blue line represents the average response expected by drift alone, with 95% confidence interval in dashed blue lines.

**Figure 3:**
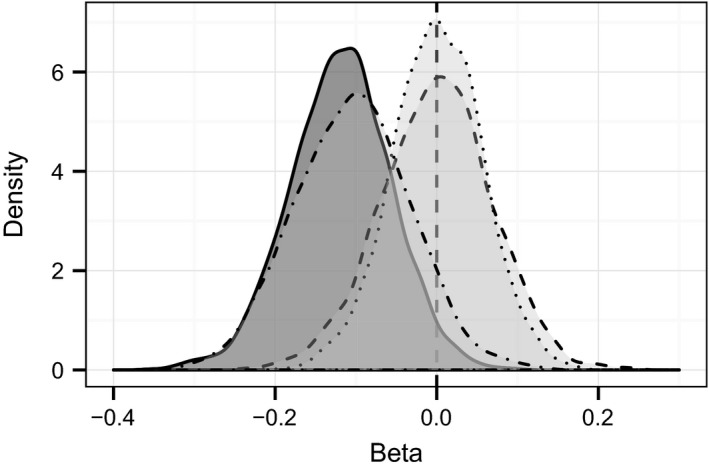
Posterior density plots for the slopes in mean estimated breeding values of male horn length and predicted change in estimated breeding value according to different models of evolutionary change for bighorn sheep cohorts born at Ram Mountain from 1973 to 1996. The dark filled distribution with solid line represents the posterior distribution of slopes in mean cohort breeding values (ße) for male horn length. The distribution with dot‐dashed line represents predicted annual evolutionary response according to the secondary theorem of selection. The distribution with dotted line represents predicted change according to simulation of stasis. The distribution with dashed line represents predicted changes due to drift according to simulation of random breeding values.

Temporal changes in mean phenotypic values over 39 years differed between traits (Fig. 1). A temporal change in EBV was also observed (Fig. 2). During the hunted period from 1973 to 1996, the EBV of male horn length declined significantly (ß = −0.119; CI = −0.248, −0.006). Similarly, genetically correlated traits also appeared to decline. Female horn length breeding value declined with a slope of −0.027 (CI = −0.063, 0.013), while EBV for male horn base had a slope of −0.030 (CI = −0.076, 0.019). Unlike male horn length, the breeding value of female horn base appeared to increase, with a slope of 0.005 (CI = −0.008, 0.016). We then compared observed changes in EBV for male horn length to those expected under various models of evolutionary change (Table S2; Fig. 3). The observed temporal change in estimated breeding value differed significantly from 0 (Pr[ße < 0] = 0.974). While observed EBV did not differ significantly from that predicted by other models, the probability of declining more than expected by drift alone (Pr[ße < ßr]) was 0.901. The observed temporal change in EBV was most similar to that predicted by the secondary theorem of selection (expected change per generation of −0.76, Fig. 3) with a posterior difference of 0.016, while the posterior differences of other models of evolution ranged from 0.117 to 0.120 (Table S2). Similarly, for female horn length, observed trends were most similar to those predicted by the secondary theorem of selection (expected change per generation of −0.10) with a posterior difference of 0.013. Other models of evolution all had similar differences of 0.027. The probability of declining more than expected by drift alone (Pr[ße < ßr]) was 0.816. For male horn base, observed trends were also most similar to those predicted by the secondary theorem of selection (expected change per generation of −0.13) with a posterior difference of 0.013. Other models of evolution all had similar differences of 0.030. The probability of declining more than expected by drift alone (Pr[ße < ßr]) was 0.796. Finally, for female horn base, all models were similar. The predicted response according to the secondary theorem of selection (expected change per generation of 0.005) had a difference of −0.004. Other models had differences of −0.005.

After the near‐cessation of hunting in 1996, average EBV remained stable or showed a weak tendency to increase, with slopes of 0.053 (CI: −0.174, 0.282) and 0.021 (CI: −0.054, 0.104) for horn length of males and females respectively. For horn base, EBVs after the change in regulations had slopes of 0.032 (CI: −0.056, 0.137) and −0.006 (CI: −0.029, 0.018) for males and females respectively. The probabilities that the slope in EBV of male and female horn length increased after the change in hunting regulations were 0.894 and 0.847 respectively. Similarly, the probabilities that the slope in EBVs of male and female horn base increased after the change in regulations were 0.866 and 0.226. Unfortunately, we could not compare observed changes in EBVs for male horn length to those expected under various models of evolutionary change for the not‐hunted period. Because of the shorter period and a smaller population size, we did not have adequate statistical power to estimate predicted responses to selection. A comparison of observed temporal trends in EBV to alternative models of evolutions such as drift or stasis suggested that all models were quite similar (Table S2).

Results for the univariate animal models were qualitatively similar to the multivariate model presented here. For the male‐only model, the posterior probabilities of declining more than expected by drift (Pr[ße < ßr]) were 0.874 and 0.629 for horn length and horn base. Posterior probabilities of declining more than expected by drift changed to 0.560, and 0.637 respectively after the change in hunting regulations (for complete results, see Table S3). For the two‐sex model, the posterior probabilities that breeding values declined more than expected by drift (Pr[ße < ßr]) were 0.985 and 0.503 for horn length of males and females, respectively. These probabilities were 0.745 and 0.582 for horn base of males and females (Table S4). Changes in breeding value had a low probability of being steeper than expected from drift after 1996 (0.560 and 0.637 for horn length, and horn base of males; 0.543 and 0.555 for the same traits in females).

## Discussion

We assessed whether temporal genetic trends in wild bighorn sheep were consistent with evolutionary changes expected from selective pressures acting on traits targeted or not targeted by trophy hunting. Using a 39‐year dataset, we expand upon previous results (Coltman et al. 2003), using a statistical approach (Hadfield et al. 2010) that is robust to biases likely affecting earlier estimates of breeding values. A model including a term for the random effect of year, as suggested by Postma (2006) confirms a statistically significant negative trend in EBV for male horn length during a period of intense harvest. Hadfield et al. (2010) suggested an even more conservative test, comparing the observed change in breeding value to simulated changes that may occur through genetic drift. The observed decline in male horn length breeding value had a probability of 90.1% of being greater than expected from drift alone, although this probability varied when using simpler univariate models (87.4% and 98.5% depending on the univariate animal model used; Tables S1–S2). The decline in breeding value had a very high probability of being greater than that expected under genetic drift in a univariate model that included information on phenotype and pedigree from both sexes but did not include genetic covariance with other traits (Table S2).

The decline in male horn length breeding values appeared to stop when hunting pressure was greatly reduced. While horn length declined during the hunting period, female horn base, a trait not subjected to trophy hunting and with low genetic correlation (0.189) to male horn length (Table 2), did not decline, supporting the contention that the decline in horn length was partly due to artificial selection. Further, female horn length and male horn base, traits genetically correlated to male horn length but not under selection, showed responses similar to male horn length. Overall, these results provide compelling evidence of a response to artificial selection while refuting the hypothesis that the observed changes were entirely caused by changes in environment. Our study population is small (average of 28.5 adult rams, yearly range 8–61) and after the hunting regulations were changed it declined partly through cougar (*Puma concolor*) predation (Festa‐Bianchet et al. 2006), averaging 17 rams. Therefore, drift may play a substantial role in changes in allele frequencies and fluctuations in breeding values over time.

Traill et al. (2014) suggested that all phenotypic changes in mass observed at Ram Mountain were due to demographic changes in response to hunting. Our analyses of horn length, however, support the result of Coltman et al. (2003) and suggest that observed changes in horn length were due to an evolutionary response to artificial selection. The difference between these studies can be explained in two ways. First, the simulations presented by Traill et al. (2014) were based on body mass. Although horn length and body mass have a moderate genetic correlation (0.48, Poissant et al. 2012), mass is not a direct target of trophy hunting. More importantly, the inheritance function in Traill et al. (2014) links parent and offspring phenotype solely upon the relationship between parental mass at conception and offspring mass at weaning: it does not allow large fathers to produce offspring that grow to become large adults (Hedrick et al. 2014; Chevin 2015) despite strong heritability of adult mass in this population (Poissant et al. 2012). The ‘inheritance’ function is nearly zero for father‐offspring, while the mother‐offspring function explains only about 5% of the variance in weaning mass (Festa‐Bianchet and Jorgenson 1998; Réale et al. 1999).

Between 1973 and 1996, the horn length of bighorn rams on Ram Mountain declined by nearly 30% (Coltman et al. 2003). It has since recovered by about 13%. When the artificial selection stopped, EBV did not increase, but there was a phenotypic increase in horn length. The very low population density in the last 15 years may have contributed to the non‐genetic increase in mean age‐corrected horn length, which remains smaller than 30–40 years ago (Fig. 1). Environmental factors such as population density and weather play important roles in horn growth (Jorgenson et al. 1993; Festa‐Bianchet et al. 2014). For example, a doubling of population size at Ram Mountain contributed to a decline in ram horn length, which, however, remained stable during an earlier period of experimental population control through ewe removals (Jorgenson et al. 1998). Therefore, it is important to adequately partition environmental and genetic phenotypic changes. Including both cohort and year in the animal model should control for both long‐ and short‐term effects of these variables on the phenotype (Wilson et al. 2010).

It seems reasonable to expect that strong artificial selection on heritable traits may lead to evolutionary changes (Garland and Rose 2009). A study on 74 domestic sheep (*Ovis aries*) breeds found strong genetic signals of selection for the absence of horns and for other traits such as body size, reproduction and pigmentation (Kijas et al. 2012). Evidence for the evolutionary effects of selective hunting in wild terrestrial species, however, remains scarce and controversial (Mysterud 2011). We suggest that evidence is scarce partly because it requires detailed long‐term data on genotypes, phenotypes, vital rates, population fluctuations, harvest pressure and environmental changes in harvested populations. Most longitudinal studies of wild vertebrates that have collected these data have been conducted on unharvested populations. There is abundant support for artificial selection in commercially‐exploited fish and recent studies provide evidence of a genetic response to that selection over a few generations (Swain et al. 2007; van Wijk et al. 2013). Therefore, it should not be surprising to find an effect of artificial selection over about 3–4 generations of bighorn sheep, given that rams with 4/5‐curl horn faced a 40% yearly probability of being shot and that the negative selective pressure through hunting started 2–3 years before large‐horned rams could achieve high reproductive success (Coltman et al. 2002). Long‐term phenotypic data from harvested rams support this contention by showing temporal declines in horn length in populations subject to high harvest pressure. Age‐specific horn size of Rocky Mountain bighorn rams declined in Alberta (Festa‐Bianchet et al. 2014) but not in the neighbouring province of British Columbia, where a more conservative definition of ‘legal’ ram reduces harvest pressure (Hengeveld and Festa‐Bianchet 2011). Similarly, in Stone's rams (*Ovis dalli*), early horn growth declined under intense selective harvest, but not under lower hunting pressure (Douhard et al. In Press).

Using detailed monitoring of a harvested population, we provide evidence that horn length – a trait directly targeted by trophy hunting – declined in response to intense artificial selection. The lack of evolutionary recovery in mean horn length breeding values after harvest stopped supports the hypothesis that recovery from potentially maladaptive human‐induced evolution is slow, likely because natural selective pressures are weaker than artificial ones (Swain et al. 2007; Allendorf and Hard 2009). Given the substantial economic importance of trophy hunting (Foote and Wenzel 2009) and its potential role in conservation (Leader‐Williams et al. 2001), it is critical to assess what levels of selective harvest can drive evolution in game species.

## Data archiving statement

The data used in this paper were collected over 39 years and are the infrastructure for several ongoing and planned research programs. They will be available in 10 years from the Dryad Digital Repository: http://dx.doi.org/10.5061/dryad.41d7q. They are also available upon request to anyone who wishes to collaborate with us or repeat our analysis.

## Supporting information


**Figure S1.** Harvest regulation guideline for bighorn sheep in Alberta, Canada.
**Figure S2.** Results of the sensitivity analysis showing the posterior mode with 95% Bayesian posterior interval of highest density of heritability for the multivariate animal model of (A) male horn length, (B) female horn length, (C) male horn base and (D) female horn base in bighorn sheep.
**Figure S3.** Changes in mean breeding value for cohorts of bighorn rams born at Ram Mountain between 1973 and 2011, according to male‐only univariate models.
**Figure S4.** Results of the sensitivity analysis of the male‐only univariate models showing the posterior mode with 95% Bayesian posterior intervals of highest density of heritability for the animal model of (A) male horn length (B) male horn base.
**Figure S5.** Changes in mean breeding value for bighorn sheep cohorts born at Ram Mountain between 1973 and 2011, according to two‐sex univariate models.
**Figure S6.** Sensitivity analysis of the two‐sex univariate models showing the posterior mode with 95% Bayesian posterior interval of highest density of heritability for the animal model of (A) horn length, (B) male horn base and (C) female horn base.
**Table S1.** Posterior mean and 95% credible interval for the predicted evolutionary change for one generation according to the secondary theorem of selection during the hunted period at Ram Mountain, Alberta.
**Table S2.** Posterior mean and 95% credible interval of the difference between observed change in mean estimated breeding value and alternative evolutionary models.
**Table S3.** Summary of results for the univariate animal models with data on males only.
**Table S4.** Summary of the results with the two‐sex univariate animal models.
