# Supplementary material for: Intense selective hunting leads to artificial evolution in horn size
Source: Evol Appl. 2016 Jan 29;9(4):521–30. doi: 10.1111/eva.12358 (PMC4831456; doi:10.1111/eva.12358)

1. **Intense selective hunting leads to artificial evolution in horn size**

Gabriel Pigeon, Marco Festa-Bianchet, David W. Coltman, Fanie Pelletier

# Supplementary material

1. Fig S1: Harvest regulation guideline for bighorn sheep in Alberta, Canada. A) Minimum horn size for rams harvested under 4/5 regulation. B) Minimum size for rams harvested under full curl regulation.
2.
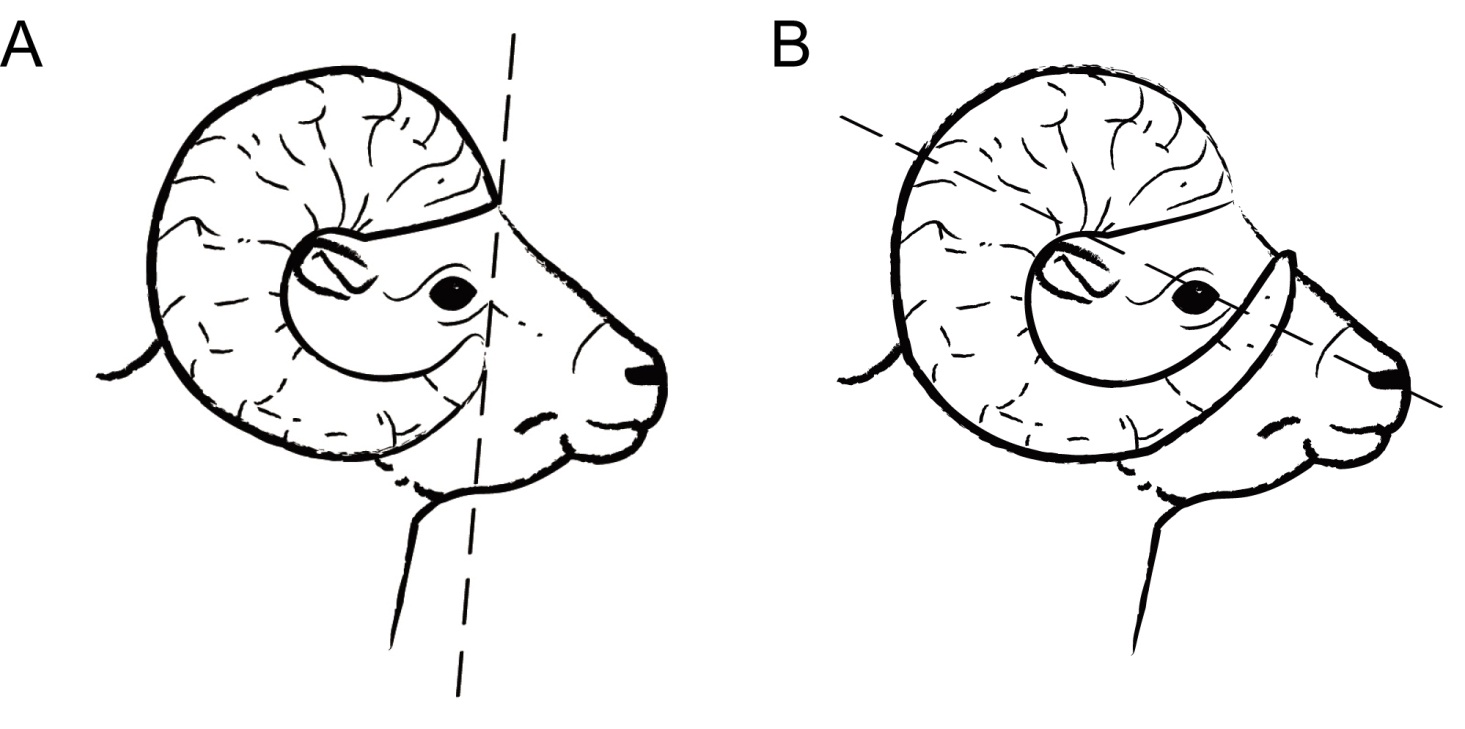


Table S1 : Posterior mean and 95% credible interval for the predicted evolutionary change for one generation according to the secondary theorem of selection during the hunted period at Ram Mountain, Alberta

|  | Hl-M | Hl-F | Hb-M | Hb-F |
| --- | --- | --- | --- | --- |
| Posterior mean | -0.757 | -0.100 | -0.126 | 0.005 |
| Lower CI | -1.845 | -0.541 | -0.495 | -0.090 |
| Upper CI | 0.178 | 0.315 | 0.317 | 0.096 |

Table S2 : posterior mean and 95% credible interval of the difference between observed change in mean estimated breeding value and alternative evolutionary models. B0 refers to a constant slope of 0, RBV refers to expected change from drift simulated by random breeding values, stasis refers to simulation of stasis and STS refers to predicted yearly changes according to the secondary theorem of selection. The alternative with the smallest difference is in bold.

| Period | Trait |  | Compared model | | | |
| --- | --- | --- | --- | --- | --- | --- |
| Hunted |  |  | B0 | RBV | Stasis | STS |
|  | Hl-M | Mean | 0.119 | 0.117 | 0.120 | **0.016** |
|  |  | Lower CI | 0.006 | -0.047 | -0.029 | -0.172 |
|  |  | Upper CI | 0.248 | 0.301 | 0.301 | 0.210 |
|  | Hl-F | Mean | 0.027 | 0.027 | 0.027 | **0.013** |
|  |  | Lower CI | -0.013 | -0.028 | -0.024 | -0.064 |
|  |  | Upper CI | 0.063 | 0.092 | 0.078 | 0.081 |
|  | Hb-M | Mean | 0.030 | 0.030 | 0.030 | **0.013** |
|  |  | Lower CI | -0.019 | -0.046 | -0.031 | -0.057 |
|  |  | Upper CI | 0.076 | 0.100 | 0.094 | 0.087 |
|  | Hb-F | Mean | -0.005 | -0.005 | -0.005 | **-0.004** |
|  |  | Lower CI | -0.016 | -0.024 | -0.021 | -0.021 |
|  |  | Upper CI | 0.008 | 0.012 | 0.009 | 0.012 |
| Not hunted | Hl-M | Mean | -0.053 | -0.054 | -0.056 |  |
|  |  | Lower CI | -0.282 | -0.463 | -0.416 |  |
|  |  | Upper CI | 0.174 | 0.328 | 0.309 |  |
|  | Hl-F | Mean | -0.021 | -0.022 | -0.021 |  |
|  |  | Lower CI | -0.104 | -0.148 | -0.127 |  |
|  |  | Upper CI | 0.054 | 0.105 | 0.105 |  |
|  | Hb-M | Mean | -0.032 | -0.031 | -0.032 |  |
|  |  | Lower CI | -0.137 | -0.190 | -0.186 |  |
|  |  | Upper CI | 0.056 | 0.114 | 0.108 |  |
|  | Hb-F | Mean | 0.006 | 0.006 | 0.006 |  |
|  |  | Lower CI | -0.018 | -0.030 | -0.029 |  |
|  |  | Upper CI | 0.029 | 0.045 | 0.040 |  |

# SI 1: Multivariate model

Figure S2: Results of the sensitivity analysis showing the posterior mode with 95% Bayesian posterior interval of highest density of heritability for the multivariate animal model of A) male horn length, B) female horn length, C) male horn base and D) female horn base in bighorn sheep. Prior *a* is a flat uninformative prior with low degree of belief (nu=1.002). Priors *b* to *h* are informative (nu=2). Prior *b* used the value of h^2^ reported by Poissant et al. (2012). Prior *c* assigned the variance equally between all variance components. Prior *d*, *e*, *f* and *g* assigned a varying proportion of the phenotypic variance to additive genetic effects (70%, 50%, 25%, 10% respectively). Dotted horizontal line shows the mode of prior *a.*
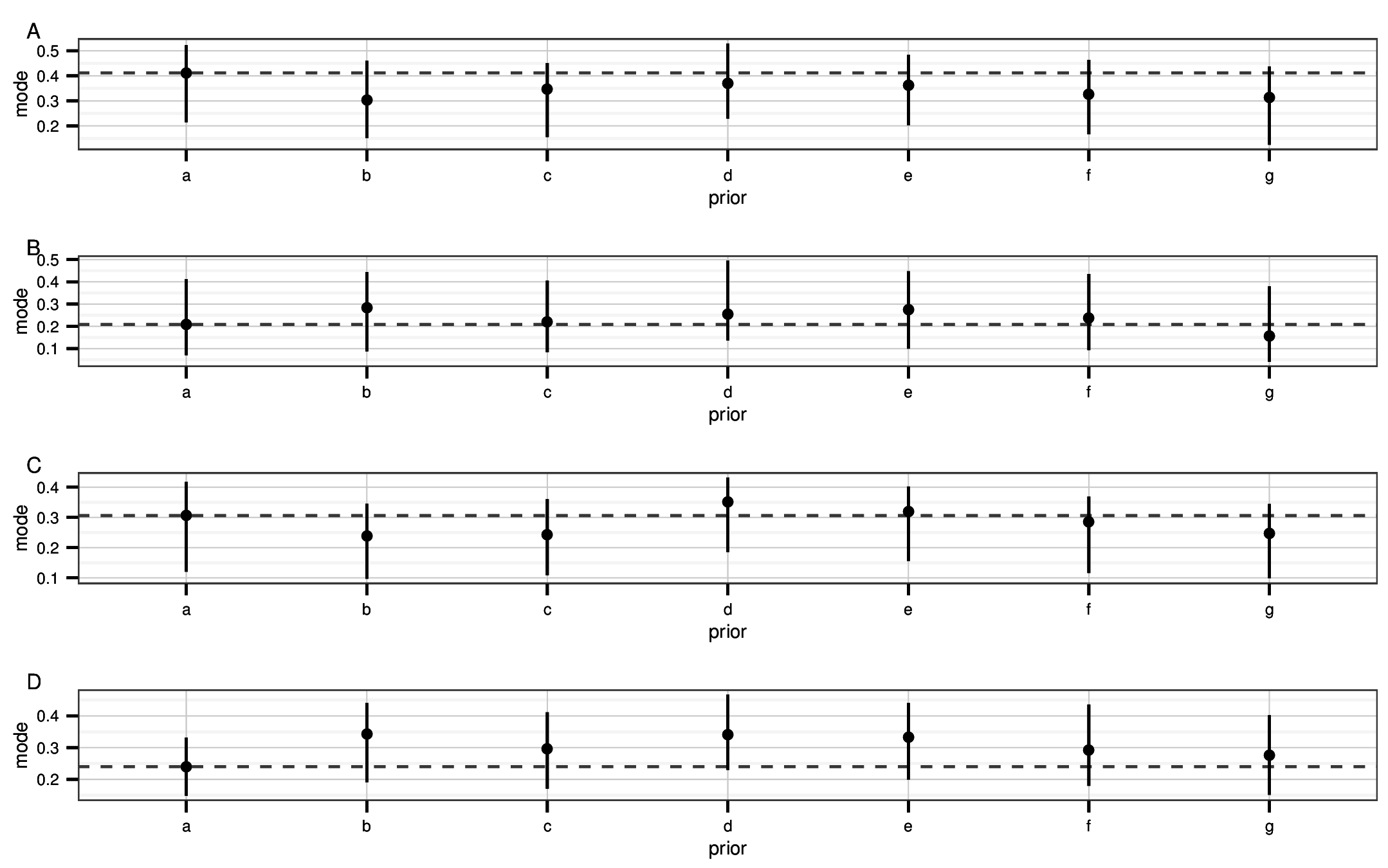


# SI 2: Male only univariate model

The “male-only” animal models were first fitted using only male phenotype. Phenotypic variance was partitioned into its components, including additive genetic variance, using a univariate animal model for each trait. The models also included sheep identity, year of measurement and cohort as random effects to assess the amount of variance due to permanent, yearly and year of birth environmental effects. Maternal identity was not included in the model since the exclusion of lambs and yearlings minimized maternal effects. Age (categorical) was included as a fixed effect.

Table S3: Summary of results for the univariate animal models with data on males only. The table shows heritability and the 95% Bayesian posterior interval of highest density for male-only univariate models of horn length and horn base. The slope of the decline (ße) in estimated breeding values through time, the probability that this decline is steeper than 0 (Pr[ße<0]) and the probability that this decline is steeper than expected by drift alone (Pr[ße<ßr]) are shown for the period subject to hunting (before 1996, hunted) and after the change in hunting regulation (after 1996, post-hunt).

|  | Heritability | | Hunted | | | Post-hunt | | |
| --- | --- | --- | --- | --- | --- | --- | --- | --- |
|  | h2 | CI | ße | Pr[ße<0] | Pr[ße<ßr] | ße | Pr[ße<0] | Pr[ße<ßr] |
| Male horn length | 0.380 | (0.188-0.549) | -0.082 | 0.946 | 0.874 | 0.023 | 0.620 | 0.560 |
| Male horn  base | 0.324 | (0.068-0.426) | -0.009 | 0.716 | 0.629 | 0.023 | 0.750 | 0.637 |

1. Figure S3: Changes in mean breeding value for cohorts of bighorn rams born at Ram Mountain between 1973 and 2011, according to male-only univariate models. Panels present the breeding values of A) horn length and B) horn base. Each grey line represents the average estimated breeding value through time for one iteration of the MCMC chain of the animal model using loess. Red dashed lines represent the posterior mean trend using linear regression for the hunted and non-hunted period. Blue line represents the average response expected by drift alone.


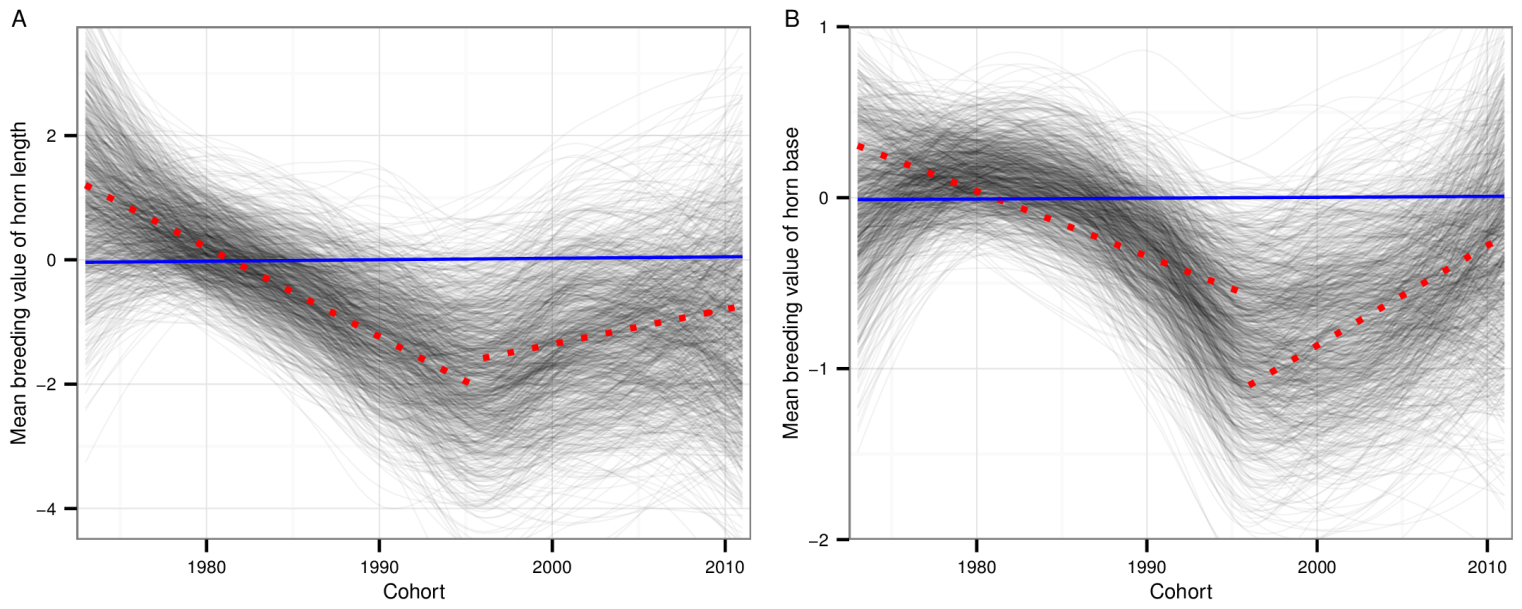


Figure S4: Results of the sensitivity analysis of the male-only univariate models showing the posterior mode with 95% Bayesian posterior intervals of highest density of heritability for the animal model of A) male horn length B) male horn base. Prior *a* is the parameter expanded prior used in further analyses and the horizontal dashed line shows the posterior mode. Prior *b* is a flat uninformative prior with low degree of belief (V=1, nu=0.002). Priors *c* to *i* are informative (nu=2). Prior *c* assigned the variance according to the posterior modes obtained from a preliminary analysis. Priors *d* and *e* used the values of h^2^ reported in Coltman et al (2005) and Poissant et al. (2012). Prior *f* assigned the variance equally between all variance components. Priors *g*, *h* and *i* assigned a large (96%), medium (60%) and low (1%) proportion of the phenotypic variance to additive genetic effects. Dotted horizontal line shows the mode of prior *a.*


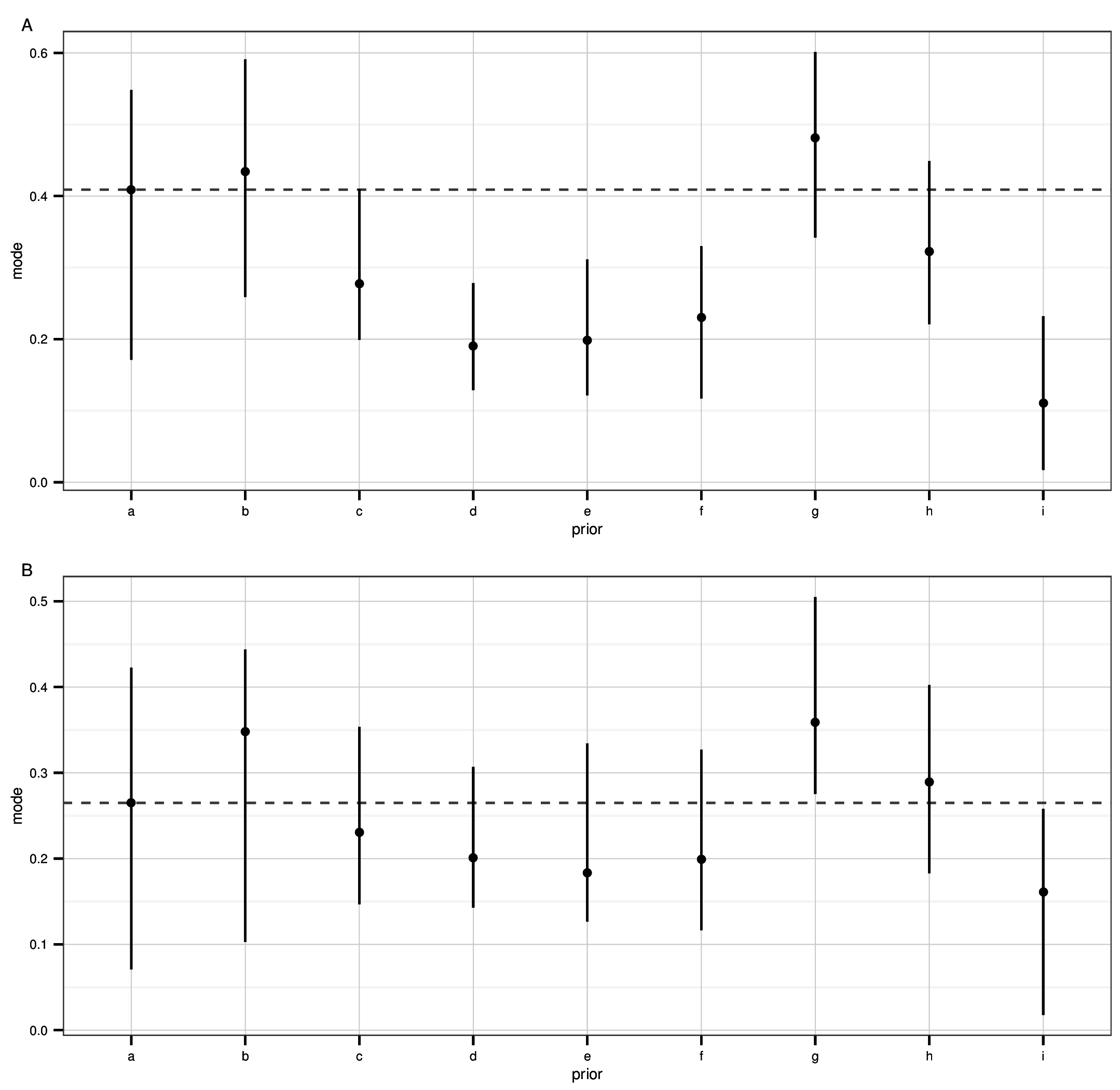


# SI 3 : Two-sex univariate model

We next fitted a “two-sex” univariate animal model including informative phenotypic information for males and females. This approach increases statistical power, revealing trends for females, which are not under direct artificial selection. Our modeling approach was similar to that described above. Given the high intersexual genetic correlation for horn length (1.00; Poissant et al. 2012), this trait can be considered the same for both sexes. Model structure was the same as described above, but also included fixed effects of sex and its interaction with age. For horn base, the intersexual genetic correlation is weak; we thus partitioned male and female horn base variance in separate models. These models were parameterised exactly as described for models including only male phenotype.

1. Table S4: Summary of the results with the two-sex univariate animal models. The table shows heritability and the 95% Bayesian posterior interval of highest density for horn length, male horn base and female horn base. The slope of the decline (ße) in estimated breeding values through time, the probability that this decline is steeper than 0 (Pr[ße<0]) and the probability that this decline is steeper than expected by drift alone (Pr[ße<ßr]) are shown for the period subject to intense hunting (before 1996, hunted) and after the change in hunting regulation (after 1996, non-hunted).

|  |  | Heritability | | | Hunted | | | Non-hunted | | | |
| --- | --- | --- | --- | --- | --- | --- | --- | --- | --- | --- | --- |
|  | Sex | h2 | CI | ße | | Pr[ße<0] | Pr[ße<ßr] | | ße | Pr[ße<0] | Pr[ße<ßr] |
| Horn length | M | 0.367 | (0.170-0.531) | -0.117 | | 1.000 | 0.985 | | 0.032 | 0.685 | 0.560 |
|  | F |  |  | 0.003 | | 0.491 | 0.503 | | 0.015 | 0.587 | 0.543 |
| Horn base | M | 0.324 | (0.068-0.426) | -0.011 | | 0.745 | 0.745 | | 0.023 | 0.750 | 0.637 |
| Horn base | F | 0.25 | (0.080-0.486) | -0.001 | | 0.582 | 0.582 | | 0.004 | 0.636 | 0.555 |

1. Figure S5: Changes in mean breeding value for bighorn sheep cohorts born at Ram Mountain between 1973 and 2011, according to two-sex univariate models. Panels present the breeding values of A-B) horn length and C-D) horn base. The left column shows result for males and the right column shows result for the females. Each grey line represents the average estimated breeding value through time for one iteration of the MCMC chain of the animal model using loess. Red dashed lines represent the posterior mean trend using linear regression for the hunted and non-hunted period. Blue line represents the average response expected by drift alone.
2.
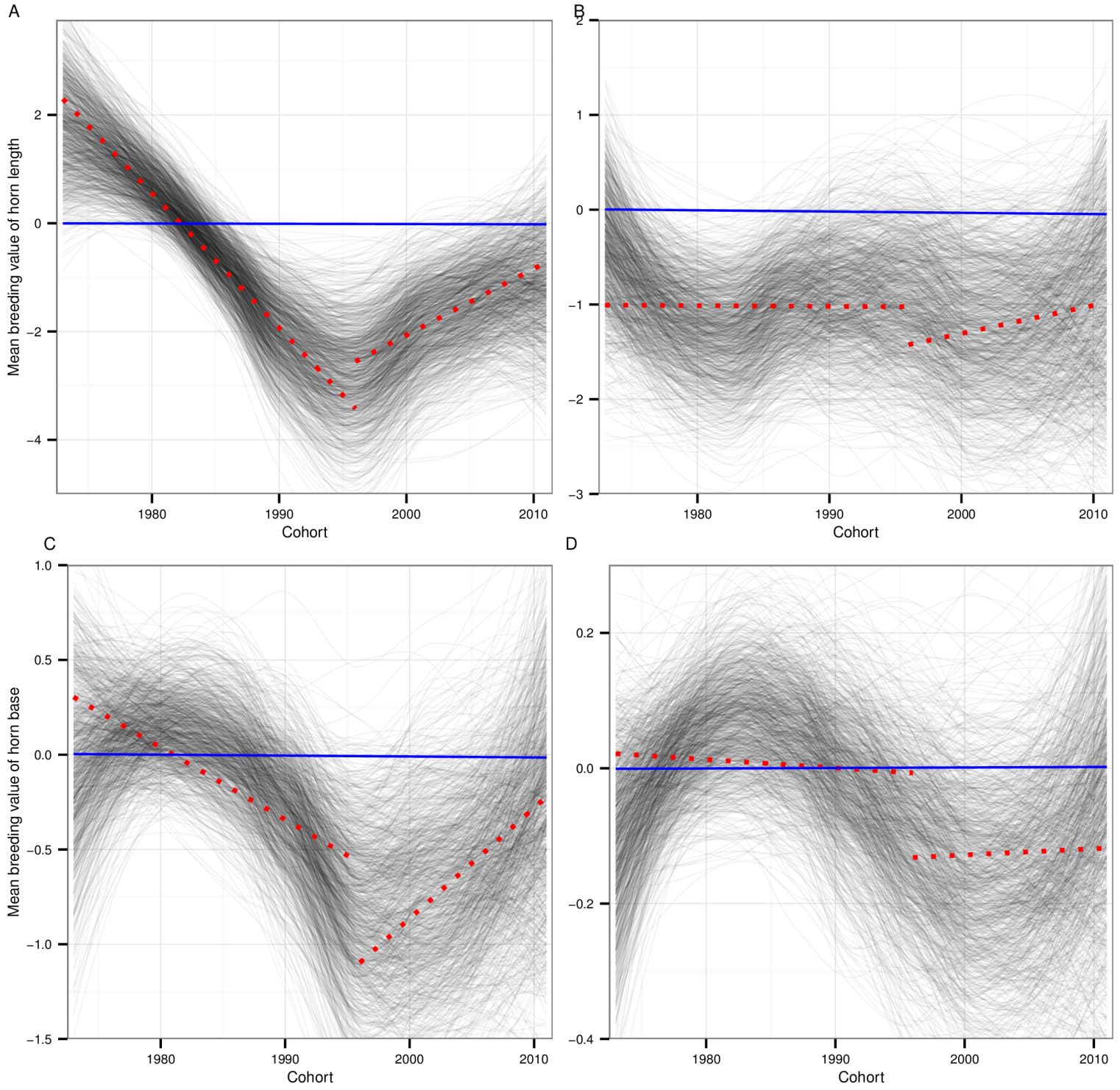


Figure S6: Sensitivity analysis of the two-sex univariate models showing the posterior mode with 95% Bayesian posterior interval of highest density of heritability for the animal model of A) horn length, B) male horn base and C) female horn base. Prior *a* is the parameter expanded prior used in further analyses and the horizontal dashed line shows the posterior mode. Prior *b* is a flat uninformative prior with low degree of belief (V=1, nu=0.002). Priors *c* to *i* are informative (nu=2). Prior *c* assigned the variance according to the posterior modes obtained from a preliminary analysis. Priors *d* and *e* used the value of h^2^ reported in Coltman et al (2005) and Poissant et al. (2012). Prior *f* assigned the variance equally between all variance components. Priors *g*, *h* and *i* assigned a large (96%), medium (60%) and low (1%) proportion of the phenotypic variance to additive genetic effects.


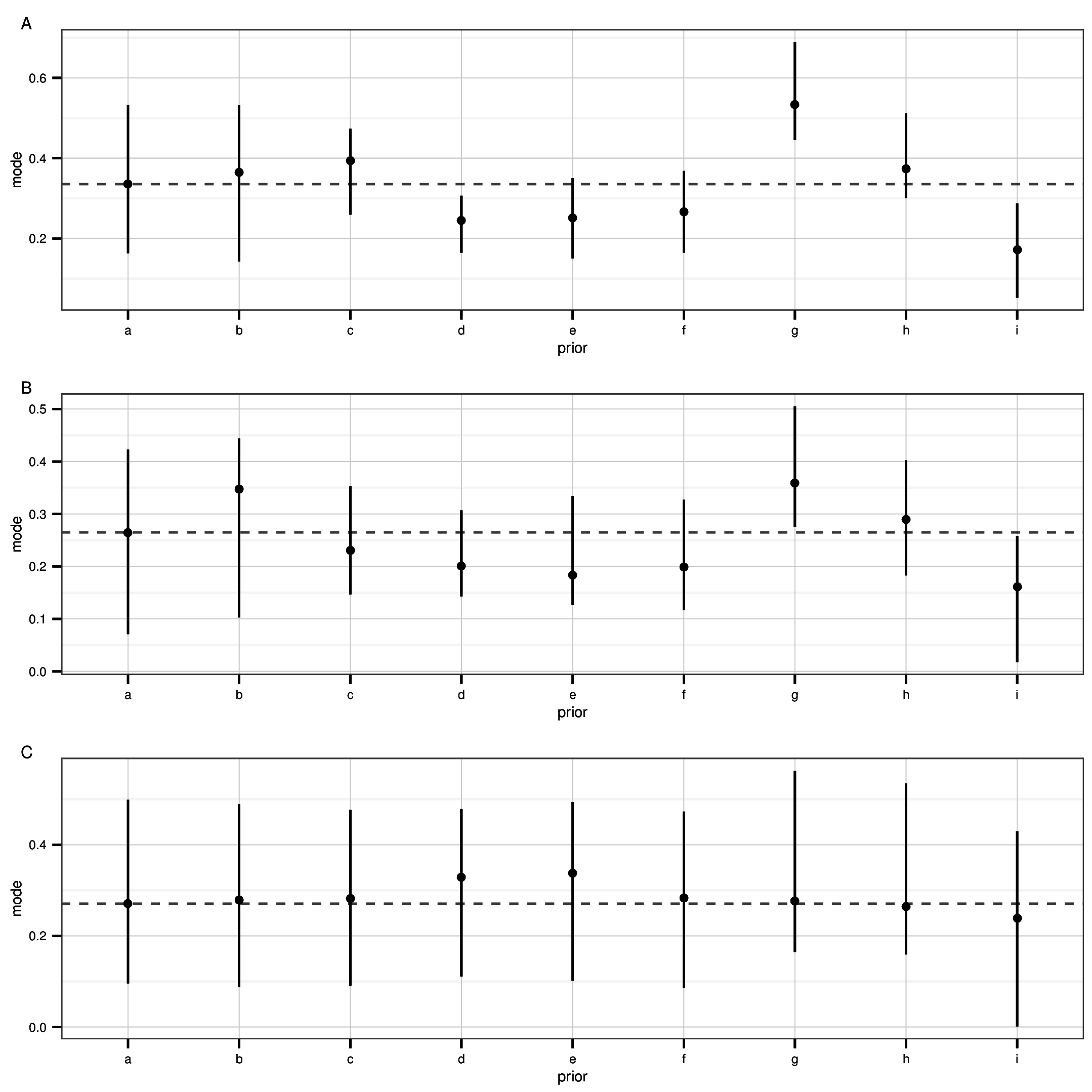

Supplement: Supplementary file 1 — Figure S1. Harvest regulation guideline for bighorn sheep in Alberta, Canada. Figure S2. Results of the sensitivity analysis showing the posterior mode with 95% Bayesian posterior interval of highest density of heritability for the multivariate animal model of (A) male horn length, (B) female horn length, (C) male horn base and (D) female horn base in bighorn sheep. Figure S3. Changes in mean breeding value for cohorts of bighorn rams born at Ram Mountain between 1973 and 2011, according to male‐only univariate models. Figure S4. Results of the sensitivity analysis of the male‐only univariate models showing the posterior mode with 95% Bayesian posterior intervals of highest density of heritability for the animal model of (A) male horn length (B) male horn base. Figure S5. Changes in mean breeding value for bighorn sheep cohorts born at Ram Mountain between 1973 and 2011, according to two‐sex univariate models. Figure S6. Sensitivity analysis of the two‐sex univariate models showing the posterior mode with 95% Bayesian posterior interval of highest density of heritability for the animal model of (A) horn length, (B) male horn base and (C) female horn base. Table S1. Posterior mean and 95% credible interval for the predicted evolutionary change for one generation according to the secondary theorem of selection during the hunted period at Ram Mountain, Alberta. Table S2. Posterior mean and 95% credible interval of the difference between observed change in mean estimated breeding value and alternative evolutionary models. Table S3. Summary of results for the univariate animal models with data on males only. Table S4. Summary of the results with the two‐sex univariate animal models. [file EVA-9-521-s001.docx]
